# Supplementary material for: A Wireless Autonomous Real-Time Underwater Acoustic Positioning System
Source: Sensors (Basel). 2022 Oct 26;22(21):8208. doi: 10.3390/s22218208 (PMC9654034; doi:10.3390/s22218208)
Supplement: Supplementary file 1 [file sensors-22-08208-s001.zip › supplementaries/s3.pdf]

Performance of off-grid PV system

PVGIS-5 estimates of solar electricity generation

Provided inputs

Latitude/Longitude:	42.605,9.291	Slope angle:	0 °
Horizon:	Calculated	Azimuth angle	0 °
Database used:	PVGIS-SARAH2	**Simulation outputs**	
PV installed:	35 Wp	Percentage days with full battery:	98.11 %
Battery capacity:	120 Wh	Percentage days with empty battery:	0 %
Cutoff limit:	20 %	Average energy not captured:	85.29 Wh
Consumption per day:	15.6 Wh	Average energy missing:	0 Wh

Outline of horizon at chosen location:

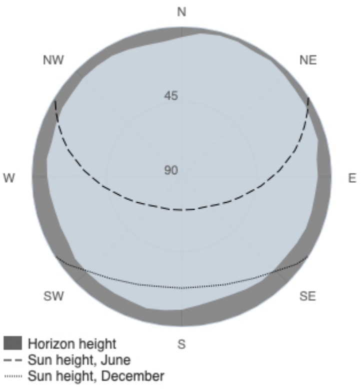

Power production estimate for off-grid PV:

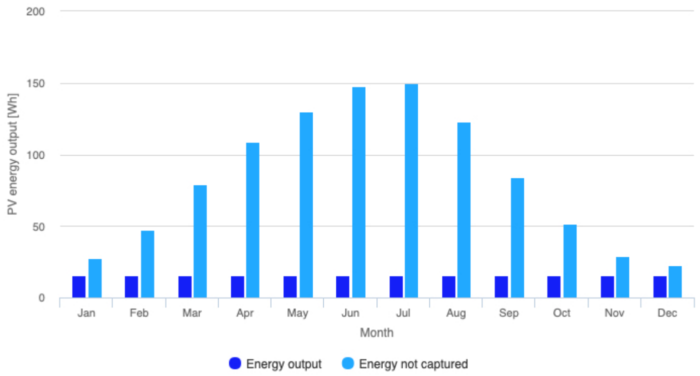

Battery performance for off-grid PV system:

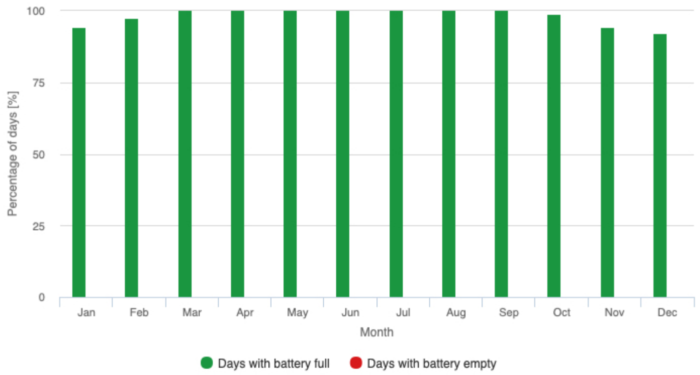

Probability of battery charge state at the end of the day:

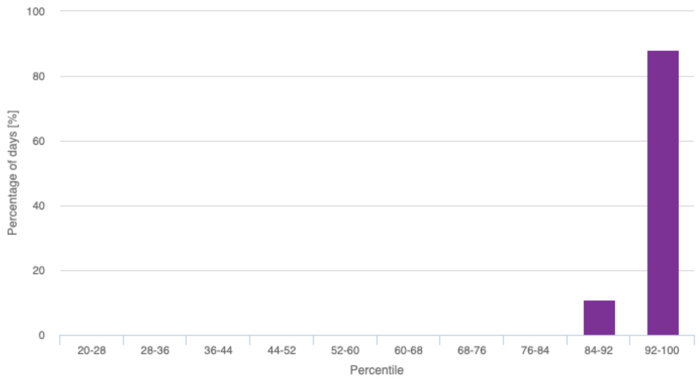

Monthly average performance

Month	E_d	E_l	f_f	f_e
January	15.6	27.7	94.3	0.0
February	15.6	47.6	97.5	0.0
March	15.6	79.3	100.0	0.0
April	15.6	108.5	100.0	0.0
May	15.6	129.7	100.0	0.0
June	15.6	147.8	100.0	0.0
July	15.6	149.8	100.0	0.0
August	15.6	123.1	100.0	0.0
September	15.6	84.0	100.0	0.0
October	15.6	51.7	98.8	0.0
November	15.6	29.1	94.4	0.0
December	15.6	22.9	92.2	0.0

E_d: Average energy production per day [Wh/day].
E_l: Average energy not captured per day [Wh/day].
f_f: Percentage of days when battery became full [%].
f_e: Percentage of days when battery became empty [%].

Cs	Cb
20-28	0.0
28-36	0.0
36-44	0.0
44-52	0.0
52-60	0.0
60-68	0.0
68-76	0.0
76-84	0.0
84-92	11.0
92-100	88.0

Cs: Charge state at the end of each day [%].
Cb: Percentage of days with this charge state [%].

The European Commission maintains this website to enhance public access to information about its initiatives and European Union policies in general. Our goal is to keep this information timely and accurate. If errors are brought to our attention, we will try to correct them. However, the Commission accepts no responsibility or liability whatsoever with regard to the information on this site.

It is our goal to minimise disruption caused by technical errors. However, some data or information on this site may have been created or structured in files or formats that are not error-free and we cannot guarantee that our service will not be interrupted or otherwise affected by such problems. The Commission accepts no responsibility with regard to such problems incurred as a result of using this site or any linked external sites.

For more information, please visit https://ec.europa.eu/info/legal-notice_en

Joint Research Centre

PVGIS ©European Union, 2001-2022.
Reproduction is authorised, provided the source is acknowledged, save where otherwise stated.

Report generated on 2022/07/06
